# Luteolin activates M2 macrophages and suppresses M1 macrophages by upregulation of hsa_circ_0001326 in THP-1 derived macrophages

**DOI:** 10.1080/21655979.2022.2036897

**Published:** 2022-02-14

**Authors:** Benxin Gong, Ying Zheng, Jiahua Li, Huafeng Lei, Kexin Liu, Jingyun Tang, Yanrong Peng

**Affiliations:** University of Chinese Academy of Sciences, Shenzhen Hospital East Hospital Pediatrics, Shenzhen City, China

**Keywords:** Luteolin, hsa_circ_0001326, macrophages polarization, THP-1 derived macrophages

## Abstract

Asthma is accompanied by inflammatory progression. Macrophages are a major type of cells to response inflammation caused by different type of factors by polarized into specific phenotypes. Luteolin and glycyrrhizic acid exert protect role in asthma; however, their role in THP-1 derived macrophages polarization whether through regulating the expression of hsa_circ_0001326 is still unknown. The effect of luteolin and glycyrrhizic acid on THP-1 derived macrophages polarization were evaluated using qRT-PCR, Western blotting, and ELISA assay. The function of hsa_circ_0001326 on macrophages polarization in luteolin treated THP-1 derived macrophages were assessed after silence of hsa_circ_0001326. And the expression of its’ potential downstream gene, including hsa-miR-136-5p and ubiquitin-specific protease 4 (USP4), were detected using qRT-PCR and Western blot analysis. Furthermore, the potential mechanism of hsa_circ_0001326 were validated using rescue experiment. Results showed that luteolin promoted M2 polarization and inhibited M1 polarization in THP-1 induced macrophages, but glycyrrhizic acid had no these effects. Hsa_circ_0001326 expression was upregulated in luteolin treat THP-1 derived macrophages. Silence of hsa_circ_0001326 reversed the function of luteolin on macrophages polarization. In addition, hsa_circ_0001326 attenuated the inhibition effect of luteolin on hsa-miR-136-5p expression, and the promotion effect on USP4 expression. Furthermore, hsa-miR-136-5p inhibitor reversed the effect of hsa_circ_0001326 on macrophages polarization and the USP4 expression. Taken together, luteolin activates M2 macrophages and suppresses M1 macrophages by upregulation of hsa_circ_0001326. Further mechanism maybe by regulating hsa_circ_0001326 downstream gene expression, including hsa-miR-136-5p and USP4, in THP-1 derived macrophages. These findings provide a new insight for macrophage polarization under stimulation of luteolin.

## Graphical Abstract

**Figure:**
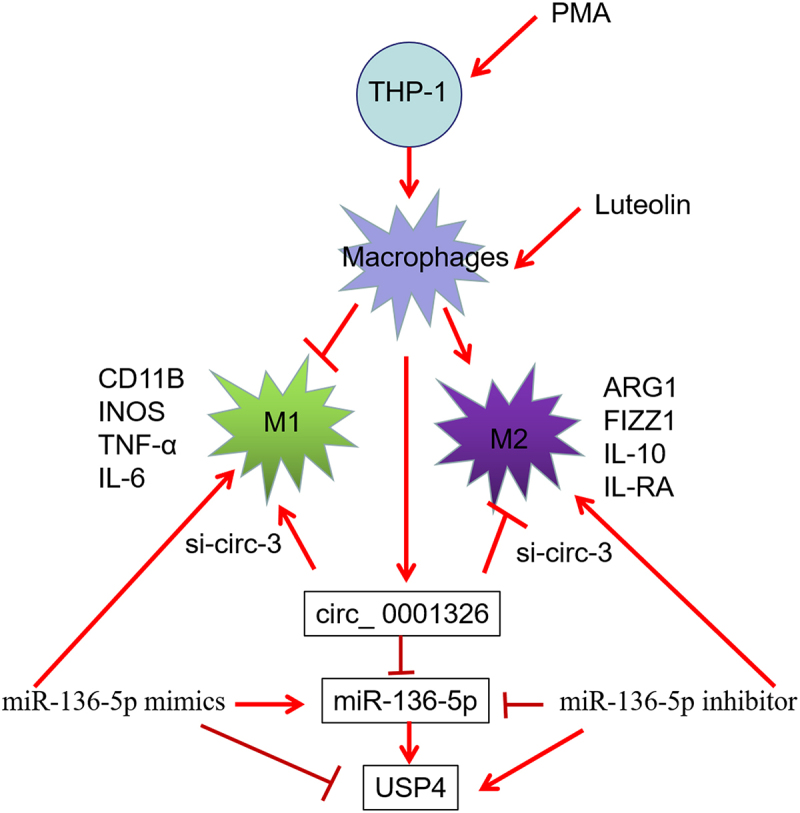

## Introduction

Asthma is accompanied by inflammatory progression and releases T helper2 (Th2) cytokines, such as interleukin 4 (IL-4) or IL-13, which were then stimulated the expression of M2 macrophage marker [1]. Macrophages are a major type of cells to response inflammation caused by different type of factors by polarized into specific phenotypes: classically activated macrophage (M1) or alternatively activated macrophage (M2) [2]. M1 macrophages often expressed pro-inflammatory genes to play an anti-microbial role, such as tumor necrosis factor alpha (TNF-α), IL-6, CD11B, and nitrogen monoxide synthase (iNOS); while M2 macrophages expressed anti-inflammatory genes for tissue regeneration, such as IL-10, IL-RA, arginase 1 (ARG1), and resistin like beta (RETNLB, also named as FIZZ1) [3,4]. At present, the human monocytic leukemia cell line THP-1 was induced into macrophage by stimulation of PMA (phorbol 12-myristate 13-acetate) can be used to investigate the potential mechanism of asthma [5], thereby our study also used this cell model for further study.

Luteolin is also named as 3’,4’,5,7-tetrahydroxyflavone, and is found in vegetables, fruits, and herbs [6]. Accumulating evidence suggests that luteolin play an important role in anti-inflammatory, anticancer, against ischemic stroke, and antiviral in vitro and in vivo experiment [7–10]. Furthermore, luteolin inhibited the intracellular expression of M2 macrophages marker in ednometriosis-associated macrophages [11], while promoted the expression of M2 macrophages marker and reduced the expression of M1 macrophages in the development of acute of lung injury, the inflammatory polarization of RAW264.7 macrophages, and angiotensin II–induced murine peritoneal macrophages [12–14]. These evidences indicated that leuteolin involved in macrophages polarization. In addition, a study showed that luteolin regulated autophagy of allergic asthma by activating PI3K/AKT/mTOR (phosphatidylinositol 3-kinase/AKT Serine/Threonine Kinase 1/Mechanistic Target of Rapamycin Kinase) pathway and inhibiting beclin-1-phosphatidylinositol 3-kinase catalytic subunit type 3 complex, which suggested that leuteolin also participate in the development of asthma [15]. Whether luteolin regulates the polarization phenotype in THP-1 induced macrophage is still unclear.

Glycyrrhizic acid is a type of natural saponin found in licorice, and can be used in candies and sweets [16]. Previous study has stated that glycyrrhizic acid had the properties of anti-oxidation, anti-inflammation, anticancer, and antiviral [17,18]. And for asthma, previous literature also validated it protect role via transforming growth factor beta 1/Smad signaling pathway or regulating the balance of T helper1 (Th1) and Th2 [19–21]. In addition, glycyrrhizic acid promoted the polarization of M1 macrophage and suppressed the polarization of M2 macrophage in murine bone marrow-derived macrophages [22]. Therefore, whether glycyrrhizic acid regulates the polarization phenotype in THP-1 induced macrophage need more study.

Circular RNAs (circRNAs) are a type of RNA with a circular structure, and have been reported to participate in different diseases acts as sponge for microRNAs (miRNA) to regulate mRNA expression, such as prostate cancer [23], osteoarthritis [23], diabetic nephropathy [24], and asthma [25,26]. Moreover, circRNAs play an important role in macrophage. For instance, inhibition of hsa_circ_0074854 suppressed exosomes-mediated macrophage M2 polarization in liver cancer [27]. circPPM1FC regulated M1 macrophage polarization in type 1 diabetes mellitus [28]. These studies indicated that circRNAs also participate in the progress of macrophage polarization.

In our study, we found a new circRNA hsa_circ_0001326 from circBase (http://www.circbase.org/), and there was no report about this circRNA at present. The parent gene is pleckstrin homology like domain family B member 2 (PHLDB2, also named as LL5beta), PHLDB2 is relates to the podosomes in macrophages and can remodel extracellular matrix (ECM) [29]. In which, ECM affect the progress of macrophage polarization [30]. Therefore, we supposed the new circRNA hsa_circ_0001326 maybe involved in the progress of macrophage polarization. To investigate whether hsa_circ_0001326 involved in THP-1 derived macrophage polarization after luteolin or glycyrrhizic acid treatment, we firstly investigate whether luteolin or glycyrrhizic acid affected the development of macrophage polarization in THP-1 derived macrophage, and found only luteolin promoted M2 polarization and inhibited M1 polarization in THP-1 induced macrophages. Then the expression of hsa_circ_0001326 and function on macrophage polarization was detected after stimulation of luteolin. Furthermore, the potential mechanism of hsa_circ_0001326 was further validated by rescue experiment. This study confirmed luteolin affect macrophage, and maybe by regulating hsa_circ_0001326 associated pathway, these findings provide a new insight for macrophage polarization under stimulation of luteolin.

## Methods and materials

### Cell culture and treatment

THP-1 cell was provided by CellCook (Guangzhou, China), and was cultured in RPMI1640 (Gibco, Grand Island, NY, USA) supplemented with 10% fetal bovine serum (Gibco) and 0.05 mM mercaptoethanol (CellCook) in a humidity incubator at 37°C. THP-1 cells were induced to macrophages using 100 ng/ml PMA (Sigma, St. Louis, MO, USA; cat: CC1904) treated for 48 h.

To investigate the function of luteolin and glycyrrhizic acid, the THP-1 cells were induced to macrophages, and then were treated with luteolin (Sigma, cat: 03600585) or glycyrrhizic acid (Sigma, cat: 1295888) at indicated concentration respectively. After 48 h, cells were collected for further analysis.

To suppress hsa_circ_0001326 expression, siRNAs was transfected into THP-1 induced macrophages following the instruction of Lipofectamine 3000 (ThermoFisher, Waltham, MA, USA). The siRNAs sequence is exhibited in Table 1.

**Table 1.** The siRNAs sequence were used in this study

siRNAs name	Sense sequence (5’-3’)	Antisense sequence (5’-3’)
si-negative control (si-NC)	GACAAGAGAAAGAGAAGCCCTT	AAGGGCTTCTCTTTCTCTTGTC
si-hsa_circ_0001326-1	GGTGAAAAGACCAAGGAGAGATT	AATCTCTCCTTGGTCTTTTCACC
si-hsa_circ_0001326-2	TGAAAAGACCAAGGAGAGACATT	AATGTCTCTCCTTGGTCTTTTCA
si-hsa_circ_0001326-3	AAGACCAAGGAGAGACAGCGTTT	AAACGCTGTCTCTCCTTGGTCTT

### Quantitative real-time polymerase chain reaction (qRT-PCR)

Cells were collected after treatment and then total RNA was isolated using TriQuick Reagent (Solarbio, Beijing, China; cat: R1100). After that, RNA (2 µg) was reversed into cDNA using HiScript III RT SuperMix for qPCR (+gDNA wiper) (Vazyme, Nanjing, China, cat: R323-01). The genes expression was detected following with the instruction of ChamQ Universal SYBR qPCR Master Mix (Vazyme, cat: Q711-02) on ABI 7500 system (ABI, Foster city, CA, USA). The primer sequence is listed in Table 2, and glyceraldehyde-3-phosphate dehydrogenase (GAPDH) was considered as internal control.

**Table 2.** Primer sequence of this study

Primer name	Sequence (5’-3’)
H-CD11B-F	GTCCAGCTTCAGGGATCCAG
H-CD11B-R	TAGTCGCACTGGTAGAGGCT
H-INOS-F	CGCATGACCTTGGTGTTTGG
H-INOS-R	CATAGACCTTGGGCTTGCCA
H-ARG1-F	ACTTAAAGAACAAGAGTGTGATGTG
H-ARG1-R	CATGGCCAGAGATGCTTCCA
H-FIZZ1-F	GTCAAAAGCCAAGGCAGACC
H-FIZZ1-R	CCAGCTGAACATCCCACGAA
H-GAPDH-F	GAGTCAACGGATTTGGTCGT
H-GAPDH-R	GACAAGCTTCCCGTTCTCAG

### Western blot analysis

Western blot analysis was performed following with previous study with a few revision [31]. In brief, the collected cells were lysed by RIPA (Beyotime Biotechnology, Shanghai, China) containing protease inhibitor (Beyotime Biotechnology). And then the protein concentration was measured using bicinchoninic acid method (Biosharp, Anhui, China; cat: BL521A). Total 20 ug protein for each sample were used for dodecyl sulfate, sodium salt – Polyacrylamide gel electrophoresis, and separated protein were then transferred onto polyvinylidene fluoride memberane. The membrane was incubated with primary antibody at 4°C overnight followed with secondary primary incubation at room temperature for 2 h. Finally, the protein bands were observed using ECL (Biosharp, cat: BL520A). The primary antibody and secondary antibody were listed as follows: CD11B (Abcam, Cambridge, MA, USA; cat: ab133357), INOS (Abcam, cat: ab178945), ARG1 (abcam, cat: ab124917), FIZZ1 (Abnova, Taipei, Taiwan; cat: PAB17997), GAPDH (Biosharp, cat: BL006B), HRP-Goat anti rabbit IgG (Biosharp, cat: BL003A), and HRP-Goat anti mouse IgG (Biosharp, cat: BL001A).

### ELISA (Enzyme Linked Immune Sorbent Assay)

Concentration of TNF-α, IL-6, IL-10, and IL-RA in cell supernatant were determined using commercial kits followed with instruction: TNF-α (Cusabio, Wuhan, China; cat: CSB-E04740h), IL-6 (Cusabio, cat: CSB-E04638h), IL-10 (Cusabio, cat: CSB-E04593h), and IL-RA (Cusabio, cat: CSB-E04629h).

### Bioinformatics analysis

To explore the potential mechanism of hsa_circ_0001326, the circular RNA interactome (https://circinteractome.nia.nih.gov/) was used to analyze the potential binding miRNAs of hsa_circ_0001326, and TargetScan Human 7.1 was used to analyze the target mRNAs of miRNAs. In the predicted mRNAs, ubiquitin-specific protease 4 (USP4) have been found play an important role in macrophages [32]. Then the potential miRNAs hsa-miR-136-5p was selected, which have predicted binding site with USP4. Finally, hsa_circ_0001326, hsa-miR-136-5p, and USP4 were selected for further study.

### Statistical analysis

Data were showed as mean ± standard deviation using Graphpad 8.0 (La Jolla, CA, USA). Difference between three groups were analyzed using one way Analysis of Variance followed with Turkey test, and *P* value less than 0.05 was considered as statistical difference.

## Results

Asthma is accompanied by inflammatory progression. Macrophages are a major type of cells to response inflammation caused by different type of factors by polarized into specific phenotypes. Luteolin and glycyrrhizic acid exert protect role in asthma. In addition, circRNAs have been reported involved in the macrophages polarization. We supposed the new circRNA hsa_circ_0001326 maybe involved in the progress of macrophage polarization. To investigate whether hsa_circ_0001326 involved in THP-1 derived macrophage polarization after luteolin or glycyrrhizic acid treatment, we firstly confirmed that only luteolin promoted M2 polarization and inhibited M1 polarization in THP-1 induced macrophages. Then we validated that luteolin affect macrophage maybe by regulating hsa_circ_0001326 and downstream gene, including miR-136-5p and USP4. These findings provide a new insight for macrophage polarization under stimulation of luteolin.

### Luteolin promoted M2 polarization and inhibited M1 polarization in THP-1 induced macrophages but glycyrrhizic acid had no these effect

**Figure 1.**
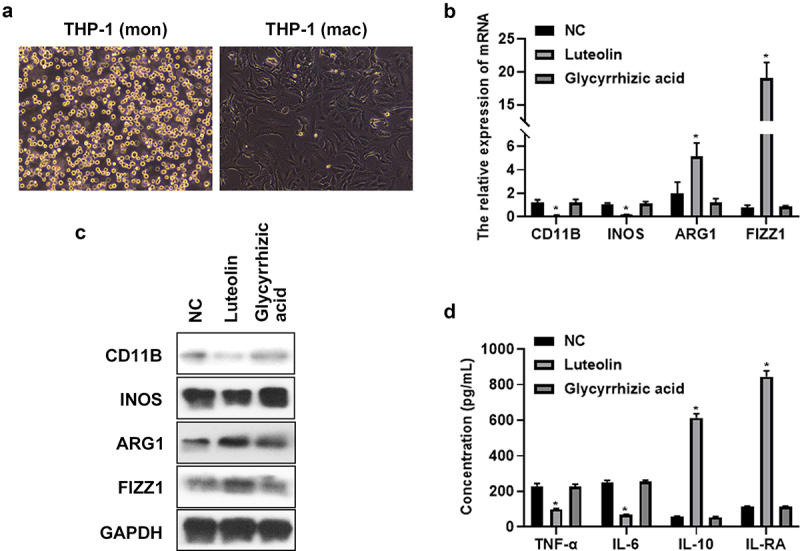
Luteolin promoted M2 polarization and inhibited M1 polarization in THP-1 induced macrophages but glycyrrhizic acid had no these effects. (a) Changes of cell morphology was observed in microscope after THP-1 induced into macrophages. (b) qRT-PCR was used to test CD11B, INOS ARG1, and FIZZ1 expression in luteolin (or glycyrrhizic acid) treated THP-1 induced into macrophages. (c) Western blot was used to evaluate CD11B, INOS ARG1, and FIZZ1 expression in luteolin (or glycyrrhizic acid) treated THP-1 induced into macrophages. (d) ELISA was used to determine the secretion of pro- and anti-inflammatory factor TNF-α, IL-6, IL-10, and IL-RA; * indicates the *p* value less than 0.05.

To investigate the function of luteolin and glycyrrhizic acid on macrophages polarization, THP-1 cells was induced into macrophages by treating with 100 ng/ml PMA for 48 h. Figure 1a shows that cells became adherent and grew with regular round or oval shape after THP-1 differentiated into macrophages. Then luteolin (100 nM) and glycyrrhizic acid (100 nM) were added into THP-1 induced macrophages for treating 48 h. The cells and cells supernatant were collected for further qRT-PCR, Western blot, and ELISA analysis. As shown in Figure 1b–c, luteolin suppressed the gene and protein expression of M1 macrophages marker (CD11B, INOS), whereas promoted the gene and protein expression of M2 macrophages marker (ARG1, FIZZ1) compared to those in NC group (THP-1 just only induced into macrophages). In addition, luteolin decreased pro-inflammatory factors (TNF-α, IL-6) secretion and promoted anti-inflammatory factor (IL-10, IL-RA) secretion (Figure 1d). However, glycyrrhizic acid had no effect on the expression of macrophages marker and inflammatory factors secretion (Figure 1b–d). The above results indicated that luteolin promoted M2 polarization and inhibited M1 polarization in THP-1 induced macrophages but glycyrrhizic acid had no these effects.

Inos: nitrogen monoxide synthase; ARG1: arginase 1; RETNLB, also named as FIZZ1: resistin like beta; TNF-α: tumor necrosis factor alpha; IL: interleukin; qRT-PCR: quantitative real-time polymerase chain reaction; ELISA: enzyme linked immune sorbent assay.

### hsa_ circ_ 0001326 inhibition reversed the effect of luteolin on macrophages polarization

**Figure 2.**
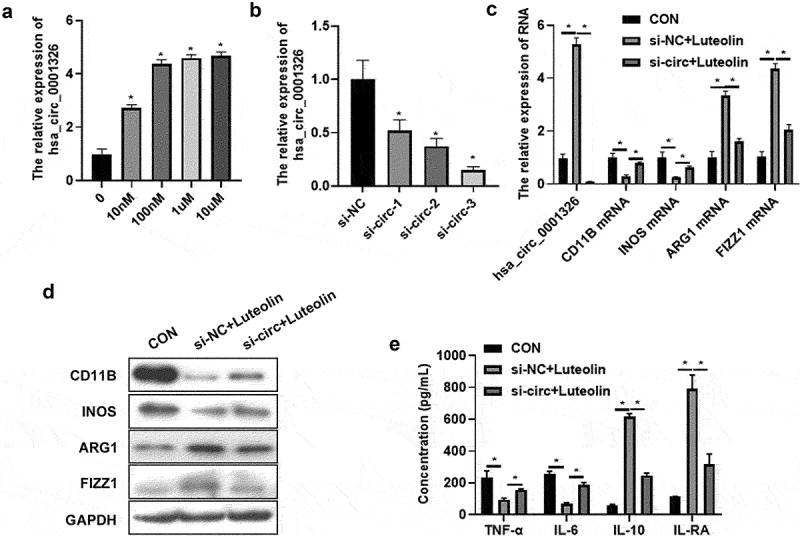
Has_ circ_ 0001326 inhibition reversed the effect of luteolin on macrophages polarization. (a) The expression of has_circ_0001326 was detected using qRT-PCR in different concentration luteolin (10 nm, 100 nm, 1 μm, and 10 μm) treated THP-1 induced macrophages. (b) The expression of has_circ_0001326 was measured using qRT-PCR after siRNAs (si-NC, si-circ-1, si-circ-2, and si-circ-3) transfected into THP-1 induced macrophages. (c) qRT-PCR and (d) Western blot analysis was used to determine the expression of CD11B, INOS, ARG1, and FIZZ1 in CON (THP-1 induced macrophages), si-NC + luteolin (si-NC was transfected into THP-1 induced macrophages for 48 h followed with luteolin treatment for another 48 h), and si-circ + luteolin group (si-circ-3 was transfected into THP-1 induced macrophages for 48 h followed with luteolin treatment for another 48 h), GAPDH was as internal reference. HAS ELISA was used to test the secretion of TNF-α, IL-6, IL-10, and IL-RA in CON, si-NC + luteolin, and si-circ + luteolin group; * indicates the p value less than 0.05.

To investigate the role of hsa_ circ_ 0001326 in luteolin treated macrophages polarization, the expression of hsa_ circ_ 0001326 was detected using qRT-PCR after different concentration treatment of luteolin. Compared to untreated group, 10 nM, 100 nM, 1 μM, and 10 μM treated group significantly promoted hsa_ circ_ 0001326 expression (Figure 2a). Then siRNAs target hsa_ circ_ 0001326 were transfected into THP-1 induced macrophages. The expression of hsa_ circ_ 0001326 was suppressed in si-circ-1, si-circ-2, and si-circ-3 group than that in si-NC group (Figure 2b). As the expression of hsa_ circ_ 0001326 decrease the most in si-circ-3 group, the si-circ-3 was selected for further experiment. After that, si-circ-3 was transfected into THP-1 induced macrophages for 48 h followed with luteolin treatment for another 48 h. Cells and cells supernatant were collected for qRT-PCR, Western blot analysis, and ELISA assay. Compared to CON group (THP-1 induced macrophages), the expression of CD11B and INOS was decreased whereas ARG1 and FIZZ1 expression was promoted si-NC + luteolin group (Figure 2c–d). In addition, the secretion of TNF-α and IL-6 was suppressed, but IL-6 and IL-RA secretion was increased (Figure 2e). However, all these effects of luteolin were reversed by si-circ-3 (Figure 2c–e). The above results suggested that hsa_ circ_ 0001326 inhibition reversed the effect of luteolin on macrophages polarization.

Inos: nitrogen monoxide synthase; ARG1: arginase 1; RETNLB, also named as FIZZ1: resist in like beta; TNF-α: tumor necrosis factor alpha; IL: interleukin; qRT-PCR: quantitative real-time polymerase chain reaction; ELISA: enzyme linked immune sorbent assay; si-NC: negative control of siRNAs; si-circ-1: siRNAs-1 target for has_ circ_ 0001326; si-circ-2: siRNAs-2 target for has_ circ_ 0001326; si-circ-3: siRNAs-3 target for has_ circ_ 0001326; GAPDH: glyceraldehyde-3-phosphate dehydrogenase.

### hsa_ circ_ 0001326 regulated the expression of hsa-miR-136-5p and USP4 in THP-1 induced macrophages after luteolin treatment

**Figure 3.**
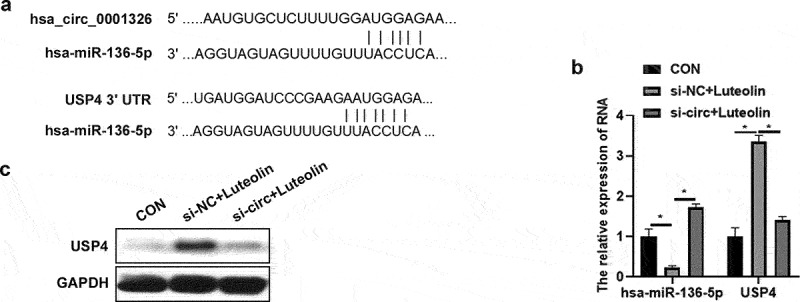
Has_ circ_ 0001326 regulated the expression of has-miR-136-5p and USP4 in THP-1 induced macrophages after luteolin treatment. (a) circular RNA interactome (https://circinteractome.nia.nih.gov/) was used to analyze the potential binding site of has-miR-136-5p and has_circ_0001326, and TargetScan Human 7.1 was used to analyze the binding site of has-miR-136-5p and USP4. (b) qRT-PCR was used to measure the expression of has-miR-136-5p and USP4 in CON (THP-1 induced macrophages), si-NC + luteolin (si-NC was transfected into THP-1 induced macrophages for 48 h followed with luteolin treatment for another 48 h), and si-circ + luteolin group (si-circ-3 was transfected into THP-1 induced macrophages for 48 h followed with luteolin treatment for another 48 h), GAPDH was as internal reference. (c) Western blot analysis was used to detect USP4 expression in CON, si-NC + luteolin, and si-circ + luteolin group; * indicates the *p* value less than 0.05.

Si-NC: negative control of siRNAs; si-circ-3: siRNAs-3 target for has_ circ_ 0001326; GAPDH: glyceraldehyde-3-phosphate dehydrogenase; USP4: ubiquitin-specific protease 4.

To explore the potential mechanism, circular RNA interactome (https://circinteractome.nia.nih.gov/) was used to analyze the potential binding miRNAs of hsa_circ_0001326, and TargetScan HμMan 7.1 was used to analyze the target mRNAs of miRNAs. In which, USP4 have been found play an important role in macrophages [32]. The binding site of USP4 and hsa-miR-136-5p, and the binding site of hsa-miR-136-5p and hsa_ circ_ 0001326 was showed in Figure 3a. So, the expression of hsa-miR-136-5p and USP4 was detected in CON, si-NC + luteolin, and si-circ + luteolin group. As showed in Figure 3b, hsa-miR-136-5p expression was promoted and USP4 expression was suppressed in si-NC + luteolin group compared to those in CON group; whereas the expression of hsa-miR-136-5p was increased, the expression of USP4 was decreased in si-circ + luteolin group than those in si-NC + luteolin group (Figure 3b–c). The above results suggested that hsa_ circ_ 0001326 regulated the expression of hsa-miR-136-5p and USP4 in THP-1 induced macrophages after luteolin treatment.

### hsa-miR-136-5p regulates USP4 expression and macrophages polarization in THP-1 induced macrophages

**Figure 4.**
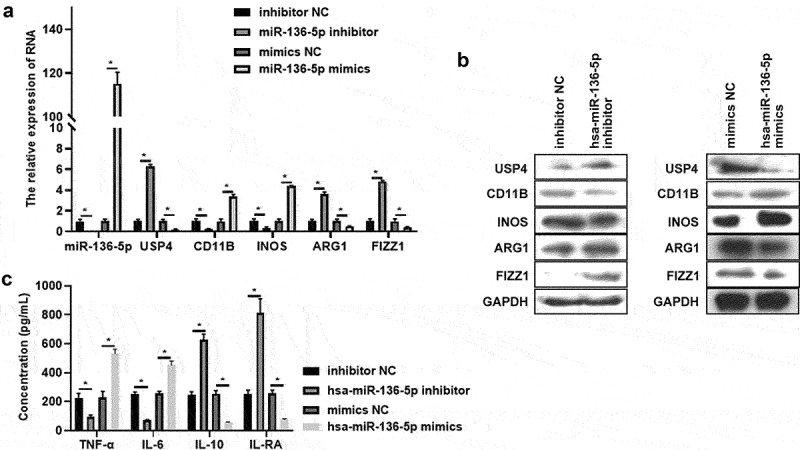
Has-miR-136-5p regulates USP4 expression and macrophages polarization in THP-1 induced macrophages. (a) The expression of has-miR-136-5p, USP4, CD11B, INOS, ARG1, and FIZZ1 in THP-1 induced macrophages with or without miR-136-5p inhibitor treatment were measured using qRT-PCR. (b) The expression of USP4, CD11B, INOS, ARG1, and FIZZ1 in THP-1 induced macrophages with or without miR-136-5p inhibitor treatment were determined using Western blot analysis. (c) The concentration of TNF-α, IL-6, IL-10, IL-RA in THP-1 induced macrophages (with or without miR-136-5p inhibitor treatment) supernatant were assessed using ELISA; * indicates the *p* value less than 0.05.

USP4: ubiquitin-specific protease 4; Inos: nitrogen monoxide synthase; ARG1: arginase 1; RETNLB, also named as FIZZ1: resistin like beta; TNF-α: tumor necrosis factor alpha; IL: interleukin; qRT-PCR: quantitative real-time polymerase chain reaction; ELISA: enzyme linked immune sorbent assay.

To further confirm the role of hsa-miR-136-5p in macrophages polarization, hsa-miR-136-5p inhibitor and mimics were synthesized and were transfected into THP-1 induced macrophages. Results exhibited that the hsa-miR-136-5p expression was significantly decreased after treated by hsa-miR-136-5p inhibitor, and its expression was increased by treating with hsa-miR-136-5p mimics (Figure 4a). The hsa-miR-136-5p’s target mRNA USP4 level was enhanced in hsa-miR-136-5p inhibitor group compared to that in inhibitor NC group, whereas hsa-miR-136-5p mimics had the reversed effect on USP4 expression (Figure 4a–b). In addition, M1 macrophages marker level (CD11B, INOS) and secretion of pro-inflammatory factors (TNF-α, IL-6) were all suppressed, M2 macrophages marker expression (ARG1 and FIZZ1) and secretion of anti-inflammatory factors (IL-10, IL-RA) were all enhanced after hsa-miR-136-5p was inhibited (Figure 4a–c). However, hsa-miR-136-5p mimics showed reverse effect (Figure 4a–c). In brief, hsa-miR-136-5p enhanced M1 polarization, and weaken M2 polarization and USP4 expression.

### hsa_ circ_ 0001326 regulates macrophages polarization via hsa-miR-136-5p/USP4 axis in THP-1 induced macrophages

**Figure 5.**
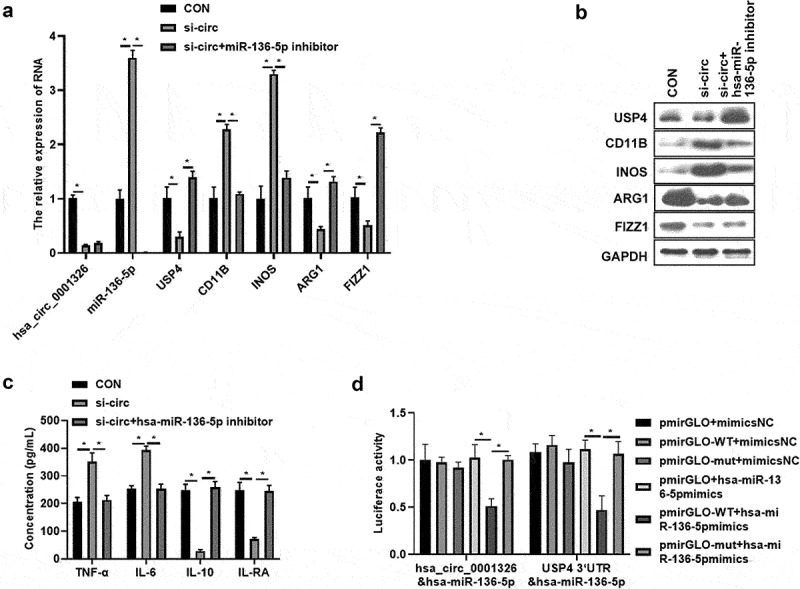
Has_ circ_ 0001326 regulates macrophages polarization via has-miR-136-5p/USP4 axis in THP-1 induced macrophages. (a) qRT-PCR was used to detect the expression of hsa-miR-136-5p, USP4, CD11B, INOS, ARG1, and FIZZ1 in CON (cotransfected si-NC and inhibitor NC), si-circ (cotransfected si-circ-3 and inhibitor NC), and si-circ+hsa-miR-136-5p inhibitor group. (b) Western blot assay was used to determine the expression of USP4, CD11B, INOS, ARG1, and FIZZ1 in CON, si-circ, and si-circ+hsa-miR-136-5p inhibitor group. (c) ELISA was used to evaluate the secretion of TNF-α, IL-6, IL-10 and IL-RA in CON, si-circ, and si-circ+hsa-miR-136-5p inhibitor group. (d) Dual luciferase reporter assay was used to further confirm the relationship between hsa_ circ_ 0001326 and hsa-miR-136-5p, the relationship between hsa-miR-136-5p and USP4; * indicates the *p* value less than 0.05.

USP4: ubiquitin-specific protease 4; iNOS: nitrogen monoxide synthase; ARG1: arginase 1; RETNLB, also named as FIZZ1: resistin like beta; TNF-α: tumor necrosis factor alpha; IL: interleukin; qRT-PCR: quantitative real-time polymerase chain reaction; ELISA: enzyme linked immune sorbent assay; si-circ-3: siRNAs-3 target for hsa_ circ_ 0001326.

To further validate the potential mechanism of hsa_ circ_ 0001326 in macrophages polarization, hsa-miR-136-5p inhibitor was cotransfected with si-circ-3 into THP-1 induced macrophages, then hsa-miR-136-5p and USP4 expression were assessed. Results showed that hsa-miR-136-5p expression was promoted (Figure 5a) and USP4 expression was suppressed (Figure 5a–b) after hsa_ circ_ 0001326 was inhibited compared to CON group (cotransfected si-NC and inhibitor NC), whereas hsa-miR-136-5p inhibitor attenuated the effect of hsa_ circ_ 0001326 on the hsa-miR-136-5p and USP4 expression (Figure 5a–b). After that, M1 macrophages marker level (CD11B, INOS), and M2 macrophages marker expression (ARG1 and FIZZ1), secretion of pro-inflammatory factors (TNF-α, IL-6), and anti-inflammatory factors (IL-10, IL-RA) were determined using qRT-PCR, Western blot analysis, and ELISA assay. As shown in Figure 5a–c, M1 macrophages marker expression and secretion of pro-inflammatory factors was enhanced, but M2 macrophages marker expression and secretion of anti-inflammatory factors was weakened by hsa_ circ_ 0001326 interference than those in CON group. However, this phenomenon was reversed by hsa-miR-136-5p inhibitor. Finally, dual luciferase reporter assay was used to further confirm the relationship between hsa_ circ_ 0001326 and hsa-miR-136-5p, the relationship between hsa-miR-136-5p and USP4. As shown in Figure 5d, luciferase activity was decreased in pmirGLO-WT (pmirGLO-hsa_ circ_ 0001326-WT or pmirGLO-USP4-WT) +hsa-miR-136-5p mimics group relative to pmirGLO+hsa-miR-136-5p mimics group or pmirGLO-mut (pmirGLO-hsa_ circ_ 0001326-mut or pmirGLO-USP4-mut) +hsa-miR-136-5p mimics group. Taken together, hsa_ circ_ 0001326 promoted M1 polarization but inhibited M2 polarization via directly regulating hsa-miR-136-5p/USP4 axis.

## Discussion

In our study, we found that luteolin promoted M2 polarization and inhibited M1 polarization in THP-1 induced macrophages, but glycyrrhizic acid had no these effects. hsa_circ_0001326 expression was promoted in luteolin treat THP-1 derived macrophages. Silence of hsa_circ_0001326 reversed the function of luteolin on macrophages polarization. In addition, hsa_circ_0001326 attenuated the inhibition effect of luteolin on hsa-miR-136-5p expression, and the promotion effect on USP4 expression. Furthermore, hsa-miR-136-5p inhibitor reversed the effect of hsa_circ_0001326 on macrophages polarization and the USP4 expression.

The incidence of asthma is high and possessed 1 of 250 deaths in the world [33]. According to the guideline provided by Global Initiative for Asthma, the majority treatment is reduction the inflammatory progress, and in the development lots of immune cells (eosinophils, neutrophils, macrophages, T-lymphocytes and mast cells) were play key roles [34]. Numerous M1 macrophages was found in asthma, thereby decreased M1 macrophages and increased M2 macrophages was one way to treat asthma, such as transglutaminase 2 [1]. Luteolin and glycyrrhizic acid have been found play anti-inflammation effect [7,17]. These suggested possibility of luteolin and glycyrrhizic acid used for treatment of asthma. In our study, luteolin made M2 polarization increased and M1 polarization decreased in THP-1 induced macrophages, consistent with previous study in the development of acute of lung injury, the inflammatory polarization of RAW264.7 macrophages, and angiotensin II–induced murine peritoneal macrophages [12–14]. Glycyrrhizic acid did not affect the macrophages polarization. The above results luteolin exert protect role in asthma maybe by regulating macrophages polarization and glycyrrhizic acid play an protect effect maybe did not through the macrophages polarization.

Then, we exhibited that hsa_circ_0001326 expression was clime up in luteolin induced THP-1 derived macrophages M2 polarization. Previous researchers demonstrated that circPPM1FC, circ_0003528, circ_0074854, and circ0048117 modulates macrophage polarization [27,28,35,36]. Hsa_circ_0001326 is a novel circRNAs, and had no any literature now. Therefore, this is the first time to exhibited that luteolin mediated macrophages polarization maybe by upregulation of hsa_circ_0001326 to play an anti-inflammation role in THP-1 derived macrophages. To further confirm the role of hsa_circ_0001326 on macrophages polarization, we knockdown the expression hsa_circ_0001326 in THP-1 derived macrophages using siRNAs followed with the evaluation of M1 and M2 polarization associated marker. And the results showed that inhibition of hsa_circ_0001326 reversed the function of luteolin on macrophages polarization. These findings were the first-time validation for confirming that hsa_circ_0001326 involved in luteolin mediated THP-1 derived macrophages polarization, and silence of hsa_circ_0001326 enhanced the development of inflammation.

As circRNAs often as a sponge for miRNAs, so we predicted hsa-miR-136-5p and USP4 were the downstream genes regulated by hsa_circ_0001326 according to bioinformation analysis from circular RNA interactome and TargetScan Human 7.1; and a study exhibited that when macrophages infected with Salmonella typhimurium, the USP4 expression was downregulated accompanied with inflammation activation [32]. In this study, reductions in USP4 level occurred in luteoin treated THP-1 derived macrophages when hsa_circ_0001326 was knockdown, indicating the activated inflammation effect, which consistent with previous study [32,37]. For miR-136-5p, its’ overexpression promoted the secretion of pro-inflammatory cytokine, and enhanced the progress of inflammation [38,39]. In our study, the expression of miR-136-5p was enhanced by silencing of hsa_circ_0001326 in luteoin treated THP-1 derived macrophages, consistent with previous study [38,39]. In addition, we validated that miR-136-5p inhibitors reversed the effect of hsa_circ_0001326 on macrophages polarization and USP4 expression. Together, we first time to propose that luteolin promoted M2 polarization and inhibited M1 polarization by regulating hsa_circ_0001326 and its downstream gene hsa-miR-136-5p and USP4.

However, this study just observed in vitro cell model, more experiment is needed to perform and validation in vivo animal model and in human cell or tissues samples. In addition, the relationship between hsa_circ_0001326 and hsa-miR-136-5p, or between hsa-miR-136-5p and USP4 are also need further verify.

## Conclusion

In brief, we found luteolin promoted M2 polarization and inhibited M1 polarization by upregulating the expression of hsa_circ_0001326, which then mediated the expression of hsa-miR-136-5p and USP4. These findings provide a new insight for asthma treatment.

## Highlights

1. Luteolin promoted M2 polarization and inhibited M1 polarization.
2. Circ_0001326 expression was upregulated in luteolin treat THP-1 derived macrophages.
3. Silence of circ_0001326 reversed luteolin’s function on macrophages polarization.
4. Luteolin promoted the expression of USP4 but inhibited miR-136-5p expression.
5. Circ_ 0001326 regulates macrophages polarization via hsa-miR-136-5p/USP4 axis.

## Abbreviations

qRT-PCR: quantitative real-time polymerase chain reaction
ELISA: enzyme linked immune sorbent assay
USP4: ubiquitin-specific protease 4
IL-4: interleukin 4
TNF-α: tumor necrosis factor alpha
iNOS: nitrogen monoxide synthase
ARG1: arginase 1; RETNLB, also named as
FIZZ1: resistin like beta
PMA: phorbol 12-myristate 13-acetate
PI3K/AKT/mTOR: phosphatidylinositol 3-kinase/AKT Serine/Threonine Kinase 1/Mechanistic Target of Rapamycin Kinase
Th2: T helper2
Th1: T helper1
circRNAs: circular RNAs
miRNA: microRNAs
GAPDH: glyceraldehyde-3-phosphate dehydrogenase
CON: THP-1 induced macrophages
si-NC: negative control of siRNAs
si-circ-1: siRNAs-1 target for hsa_ circ_ 0001326
si-circ-2: siRNAs-2 target for hsa_ circ_ 0001326
si-circ-3: siRNAs-3 target for hsa_ circ_ 0001326
